# Sleep Bruxism: Mapping Potential Direct and Indirect Risk Pathways in EPISONO Adult Population‐Based Study

**DOI:** 10.1111/joor.70128

**Published:** 2025-12-08

**Authors:** Eduardo Machado, Jessica Klöckner Knorst, Milton Maluly Filho, Monica Levy Andersen, Sergio Tufik, Cibele Dal Fabbro, Dalva Poyares

**Affiliations:** ^1^ Department of Psychobiology Universidade Federal de Sao Paulo São Paulo Brazil; ^2^ Postgraduate Program in Dental Sciences Universidade Federal de Santa Maria Santa Maria Brazil; ^3^ Faculté de Médecine Dentaire Université de Montréal Montréal Québec Canada; ^4^ Center for Advanced Research in Sleep Medicine Hôpital du Sacré‐Coeur de Montréal, CIUSSS‐NIM Montréal Québec Canada; ^5^ Centre de Recherche Institut Universitaire de Gériatrie de Montréal (CRIUGM) Montréal Québec Canada

**Keywords:** adults, comorbidities, observational study, pathway analysis, sleep, sleep bruxism, sleep disorders

## Abstract

**Aim:**

To explore the direct and indirect pathways through which sociodemographic, psychological, behavioural, and clinical factors influence sleep bruxism (SB).

**Methods:**

This cross‐sectional study was conducted with a sample of 686 adults (mean age of 50.1 years; 380 female and 306 male), from a total of 712 individuals from the Sao Paulo Epidemiological Sleep Study (EPISONO) follow performed in 2015. SB was assessed using self‐report, overnight polysomnography (PSG‐based), and combined methods. Sociodemographic, psychological, behavioural and clinical factors were assessed. Structural Equation Modelling was used to examine the pathways between potential risk factors and SB.

**Results:**

From an initial sample of 1042, 712 returned for follow‐up and 686 individuals were eligible based on the SB outcomes evaluated and having undergone PSG. The SB self‐reported prevalence was 17.1%, 30.5% presented PSG‐based SB and 7.4% in combination of methods (self‐report+PSG). Sleep bruxism (assessed by all methods) was directly associated with higher levels of insomnia and younger age. Higher socioeconomic status was directly associated with self‐reported SB, whereas PSG‐based and self‐report+PSG SB were associated with increased obstructive sleep apnea and smoking. Regarding indirect effects, elevated anxiety and depressive symptoms indirectly impacted all forms of SB via increased insomnia levels.

**Conclusions:**

Our findings highlight distinct and overlapping pathways of SB. Insomnia and younger age consistently predicted SB, while psychological factors indirectly impacted SB via insomnia. Demographic, behavioural, and clinical factors showed direct associations that varied according to the assessment method.

## Graphical abstract

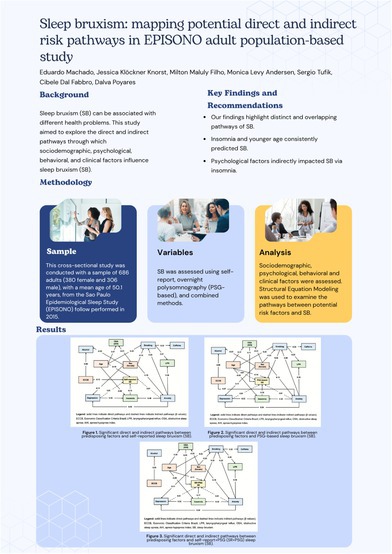

## 1 Introduction

Bruxism is a masticatory muscle activity characterised by repetitive jaw‐muscle contractions, which may include teeth grinding or clenching [1, 2]. It can occur during wakefulness (awake bruxism) or sleep (sleep bruxism, SB), and has been redefined through international consensus as a behaviour rather than a disorder [2]. SB is a masticatory muscle activity during sleep that is characterised as rhythmic (phasic) or non‐rhythmic (tonic) and is not a movement disorder or a sleep disorder [2]. Its assessment varies, with self‐reports being the most used method in epidemiological studies, although polysomnography (PSG) remains the gold standard for instrumental diagnosis [3, 4]. According to the current diagnosis standards, it should be based on self‐reports or complaints and the instrumental evaluation may add information when needed to assess the presence of another condition or for research purposes [2, 5, 6].

Prevalence estimates vary depending on the method and population studied, ranging from approximately 8% to 16% with self‐report and from 5% to 8% with PSG in population‐based samples [3, 4, 7, 8]. In an umbrella review of systematic reviews, the prevalence of SB in adults ranged from 1% to 15% [4].

The pathophysiology of SB is multifactorial and appears to involve both central nervous system (CNS) and autonomic processes, genetics and environmental factors [9, 10, 11]. Most episodes of rhythmic masticatory muscle activity (RMMA) are preceded by increased sympathetic cardiac activity, suggesting a role of the catecholaminergic and other neurochemical systems in the generation and modulation of jaw movements during sleep [12]. The aetiology of SB is complex and multifactorial, involving an interplay of predisposing, initiating, and maintaining factors [4]. Sociodemographic variables (e.g., age and sex), psychological aspects (including anxiety, stress, and depression), behavioural habits (like smoking, alcohol and caffeine intake), and clinical comorbidities (e.g., insomnia, sleep apnea and gastroesophageal reflux) have all been identified as relevant contributors [13, 14, 15, 16, 17, 18, 19, 20]. Moreover, recent studies suggest that the associations may differ depending on different phenotypes and on how SB is assessed [14, 21].

Despite these findings, most studies focus on isolated factors or use clinical samples, which limits the generalisability of the results. There is still a lack of population‐based studies examining the simultaneous influence of multiple domains, especially those exploring direct and indirect effects. Thus, this study aimed to explore the direct and indirect pathways through which sociodemographic, psychological, behavioural, and clinical factors influence SB evaluated by self‐report, PSG‐based and the combination of these methods in a population‐based sample. By adopting a comprehensive, multivariable modelling approach, this study seeks to address a gap in the literature regarding the complex associations of SB in the adult general population.

## 2 Methods

### 2.1 Study Report, Ethical Approval and Sample

This cross‐sectional study was reported following the Strengthening the Reporting of Observational Studies in Epidemiology (STROBE) guidelines. The research protocol was approved by the Research Ethics Committee of the Federal University of Sao Paulo (Approval Nos. 593/2006, 610 516/2014, and CAAE 75149723.9.0000.5505). Written informed consent was obtained from all participants before taking part in the study.

The sample used in the present study is derived from the Sao Paulo Epidemiological Sleep Study (EPISONO), a population‐based cohort designed to establish the epidemiological profile of sleep disorders in a representative, probabilistic sample of adults aged 20–80 years residing in Sao Paulo, Brazil. Sao Paulo is the most populous city in the country, with approximately 11.45 million inhabitants [22]. EPISONO includes a baseline assessment conducted in 2007 and a follow‐up in 2015. The initial sample of EPISONO 2007 involved 1042 individuals who were invited to return for the 2015 follow‐up. Of this sample, 712 participants returned and of these, 686 (about 66% of baseline) were eligible for this study based on the SB outcomes evaluated and having undergone PSG. The current analysis is based on data collected during the 2015 follow‐up. Detailed descriptions of the study design, sampling procedures, and baseline findings have been published elsewhere [3, 23].

The sample size for this study was estimated based on a standard error of 5%, a 95% confidence level, and an effect size of 0.1, considered appropriate for detecting minimal differences [24]. The calculation for pathway analysis also accounted for 12 observed variables and a statistical power of 90%. To consider potential losses to follow‐up, a 30% increase was applied, resulting in a minimum required sample size of 259 participants. As this epidemiological survey is nested in a larger study, a greater number of individuals were ultimately evaluated.

### 2.2 Data Collection and Variables

All assessments in the study were conducted by a trained and calibrated team, using questionnaires and tests previously validated in the literature. Accordingly, a variety of relevant demographic, socioeconomic, behavioural, psychological and clinical variables were collected.

The occurrence of SB, classified as present or absent, was assessed using self‐report, PSG‐based and a combined outcome. Self‐reported SB was evaluated by the question: ‘How often do you currently grind your teeth?’, with response options including: never, < 1, 1, and 2–3 times/month, 1–2, 3–6 times/week, and daily [3]. Participants were subsequently categorised as having SB (1–2 times/week or more) or not having SB (2–3 times/month or less) [3]. Participants also underwent overnight in‐lab PSG using a digital system (EMBLA S7000, Embla Systems Inc., Broomfield, CO, USA). PSG recordings followed standardised sleep investigation criteria [25]. Sensors were noninvasively attached using adhesive tape and/or collodion. Prior calibration was conducted on the right and left masseter and temporalis muscles to ensure optimal placement over the regions with the greatest muscle mass [26]. Participants who had ≥ 2 SB episodes/h of sleep were included in the group with PSG‐based SB and those with < 2 SB episodes/h of sleep were included in the group without PSG‐based SB [27]. A combined SB assessment (self‐report+PSG) was defined to identify individuals who presented SB according to both methods simultaneously. It is worth noting that the criteria adopted in this study are consistent with the core definitions and diagnostic thresholds later proposed by international consensus statements on sleep bruxism [1, 2].

Potential risk factors for SB include demographic, socioeconomic, psychological, behavioural and clinical variables. Demographic and socioeconomic data included sex (male or female), age (mean) and Economic Classification Criteria Brazil (ECCB) (mean), considered a proxy for socioeconomic level. ECCB was used to estimate the purchasing power of families, ranging from 0 to 100 points, with the higher the value on the scale, the greater the purchasing power. Psychological factors included symptoms of anxiety and depression, assessed using the Beck Anxiety Inventory (BAI) [28] and the Beck Depression Inventory (BDI) [29], respectively. Both instruments presented total scores ranging from 0 to 63, which were considered continuous variables for analytical purposes.

Behavioural factors included alcohol, smoking, and caffeine consumption. Alcohol consumption was recorded according to the number of days the individual drank per week and according to the number of cigarettes smoked per day. Caffeine consumption was assessed through a question regarding the average number of glasses or cups consumed per day, considering beverages such as coffee, black tea, and cola. All behavioural variables were used quantitatively.

Regarding clinical variables, insomnia was assessed using the Insomnia Severity Index (ISI) [30], with total scores ranging from 0 to 28. Obstructive sleep apnea (OSA) was assessed by the apnea‐hypopnea index (AHI), which evaluates the mean number of apnea‐hypopnea episodes per hour of sleep [5]. The Brazilian version of the Reflux Symptom Index (RSI) questionnaire was used to measure symptoms of laryngopharyngeal reflux (LPR), which consists of 9 items addressing numerous symptoms. Each item is evaluated on a Likert scale from 0 to 5, with total scores ranging from 0 to 45 [31, 32]. For data analysis, all clinical variables were used quantitatively.

### 2.3 Statistical Analysis

Data were analysed using STATA version 17.0 (StataCorp LLC, College Station, TX, USA). Descriptive statistics were calculated to describe the main sample characteristics.

Structural Equation Modelling (SEM) was performed to evaluate the associations between potential risk factors and SB. Three structural models were built, corresponding to each SB assessment: self‐report, PSG‐based and a combination of both (self‐report+PSG). These models estimated the magnitude and direction of both direct and indirect pathways among observed variables. All models were estimated using the Maximum Likelihood with Missing Values (MLMV) approach [33]. Model refinement considered modification indices and the removal of non‐significant paths (*p* ≥ 0.25) to improve parsimony. Model fit was assessed using the Root Mean Square Error of Approximation (RMSEA), Comparative Fit Index (CFI), and Tucker–Lewis Index (TLI). Acceptable model fit was defined as RMSEA < 0.05, and CFI and TLI > 0.90 [34]. Results are reported as standardised coefficients (β), standard errors (SE), and *p*‐values.

## 3 Results

The study sample of EPISONO 2007 involved 1042 individuals who were invited to return for the 2015 follow‐up. Of this sample, 712 returned and of these, 686 (about 66% of baseline) were eligible for this study based on the SB outcomes evaluated and having undergone PSG. Of the total sample, 55.4% were female, and the mean age was 50.1 years (SD 13.3). The mean score on the ECCB was 33.3 (SD 11.6). Regarding psychological variables, the mean scores for anxiety (BAI) and depression (BDI) were 9.4 (SD 9.0) and 9.9 (SD 9.0), respectively. Participants reported a mean daily caffeine consumption of 2.5 (SD 3.0) glasses or cups. The overall scores in the RSI were 7.0 (SD 8.0) and 6.6 (SD 5.2) in the ISI. The prevalence of SB was 17.1% based on self‐report, 30.5% PSG‐based and 7.4% in combined assessment (Table 1).

**TABLE 1:** Sample distribution considering sociodemographic, behavioural, psychological and clinical variables, Sao Paulo, Brazil (*N* = 686).

Variables	Distribution
	*N* (%)
Sex	
Male	306 (44.6)
Female	380 (55.4)

	Mean (SD)
Age (years)	50.1 (13.3)
Socioeconomic level (ECCB)	33.3 (11.6)
Anxiety (Beck Anxiety Inventory)	9.4 (9.0)
Depression (Beck Depression Inventory)	9.9 (9.0)
Alcohol consumption	0.9 (1.4)
Smoking	12.3 (9.4)
Caffeine consumption	2.5 (3.0)
LPR (Reflux Symptom Index)	7.0 (8.0)
Insomnia (Insomnia Severity Index)	6.6 (5.2)
OSA (Apnea‐Hypopnea Index)	15.2 (16.6)

Sleep Bruxism outcomes	*N* (%)
Self‐report	
Absent	567 (82.9)
Present	117 (17.1)
PSG‐based	
Absent	477 (69.5)
Present	209 (30.5)
Self‐report+PSG	
Absent	635 (92.6)
Present	51 (7.4)

Abbreviations: ECCB, Economic Classification Criteria Brazil; LPR, laryngopharyngeal reflux; OSA, obstructive sleep apnea; PSG, polysomnography; SD, standard deviation.

**FIGURE 1:**
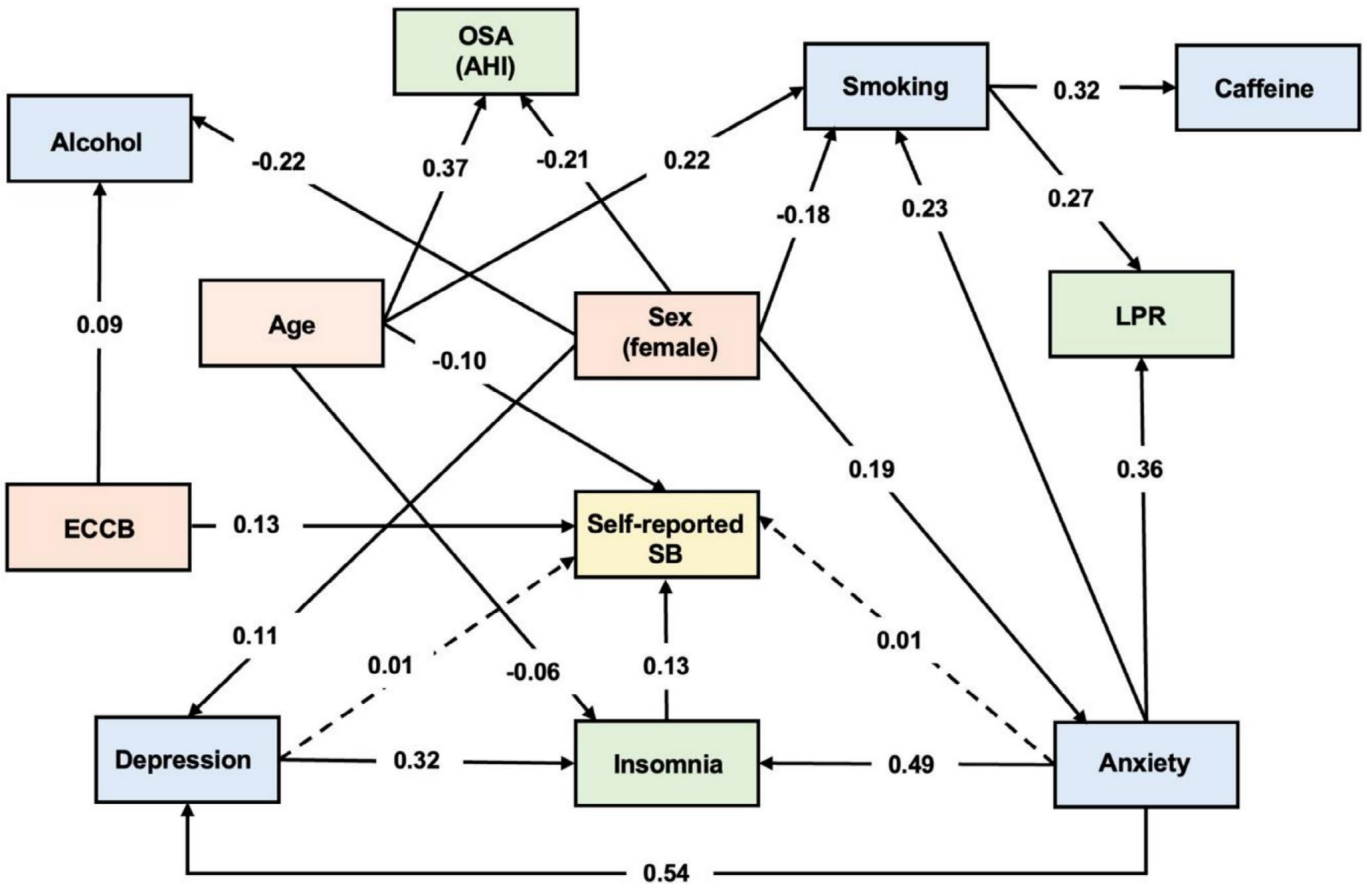
Significant direct and indirect pathways between predisposing factors and self‐reported sleep bruxism (SB). Solid lines indicate direct pathways and dashed lines indicate indirect pathways (β values). AHI, apnea‐hypopnea index; ECCB, Economic Classification Criteria Brazil; LPR, laryngopharyngeal reflux; OSA, obstructive sleep apnea.

Direct and indirect pathways with statistical significance between predictor variables and self‐reported SB are illustrated in Figure 1. Higher levels of insomnia were directly associated with increased self‐reported SB (β 0.13, SE 0.03, *p* < 0.01). Additionally, younger age (β −0.10, SE 0.03, *p* < 0.05) and higher ECCB scores (β 0.13, SE 0.03, *p* < 0.01) were directly associated with increased reports of SB. Regarding indirect effects, higher anxiety levels were associated with increased self‐reported SB via elevated insomnia and LPR levels (β 0.01, SE 0.01, *p* < 0.01). Similarly, depressive symptoms were also indirectly associated with SB through their influence on insomnia (β 0.01, SE 0.01, *p* < 0.01). The model presented a good fit as indicated by RMSEA (0.01; 0.00–0.02), CFI (1.00), and TLI (1.00) indexes.

**FIGURE 2:**
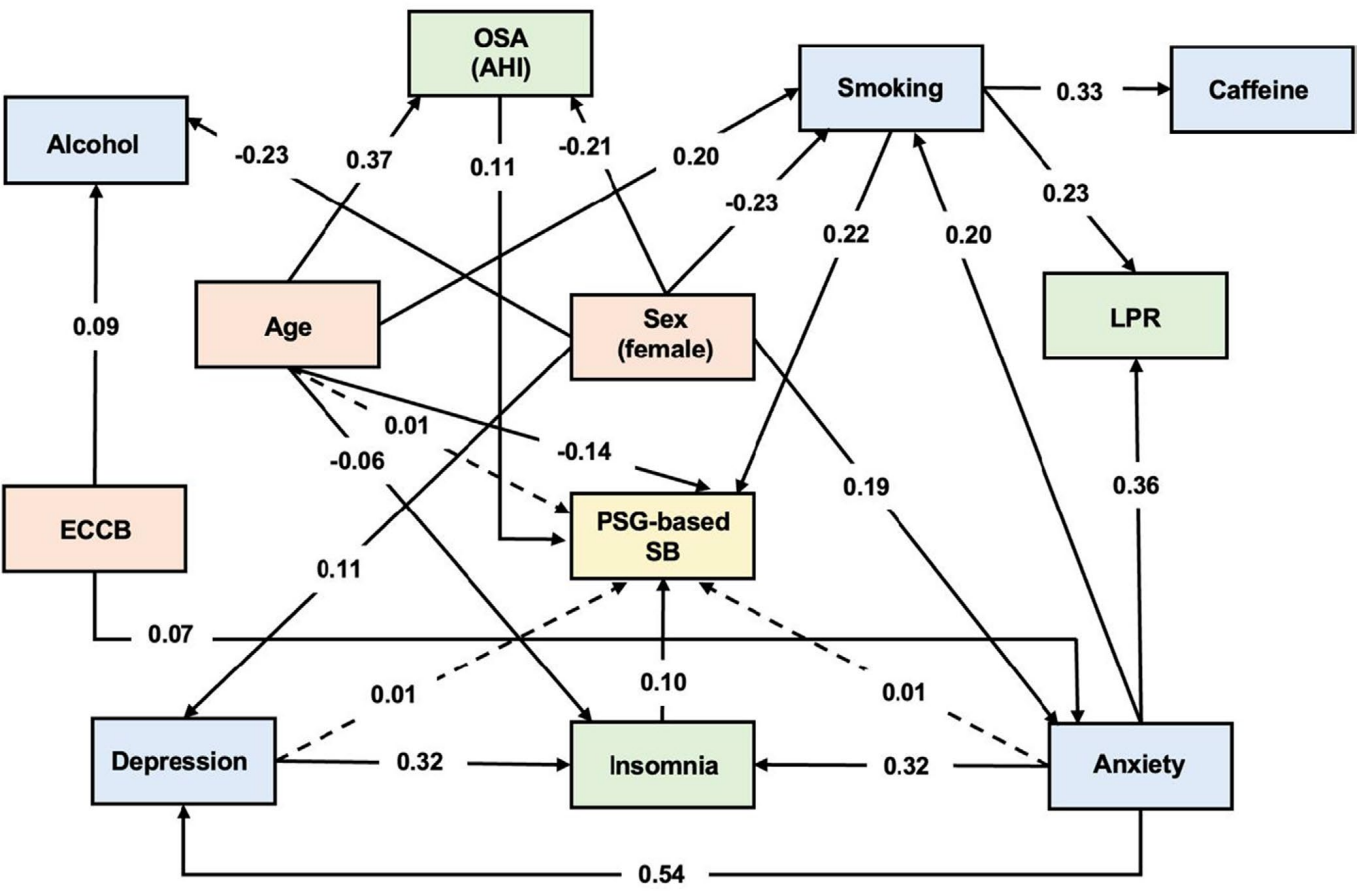
Significant direct and indirect pathways between predisposing factors and PSG‐based sleep bruxism (SB). Solid lines indicate direct pathways and dashed lines indicate indirect pathways (β values). AHI, apnea‐hypopnea index; ECCB, Economic Classification Criteria Brazil; LPR, laryngopharyngeal reflux; OSA, obstructive sleep apnea.

Figure 2 presents the significant direct and indirect pathways between predictor variables and PSG‐based SB. This model demonstrated good fit indexes: RMSEA (0.01; 0.00–0.02), CFI (1.00), and TLI (1.00). The occurrence of PSG‐based SB was directly impacted by higher levels of obstructive sleep apnea (β 0.11, SE 0.04, *p* < 0.01), insomnia (β 0.10, SE 0.03, *p* < 0.01) and smoking (β 0.22, SE 0.07, *p* < 0.01). In addition, younger age directly impacted the assessment of SB by PSG (β −0.14, SE 0.04, *p* < 0.01). Regarding indirect effects, higher levels of anxiety (β 0.01, SE 0.01, *p* < 0.05) and depression (β 0.01, SE 0.01, *p* < 0.01) indirectly impacted SB via increased insomnia. Additionally, age was indirectly associated with SB via its effects on OSA and insomnia (β 0.01, SE 0.01, *p* < 0.01).

**FIGURE 3:**
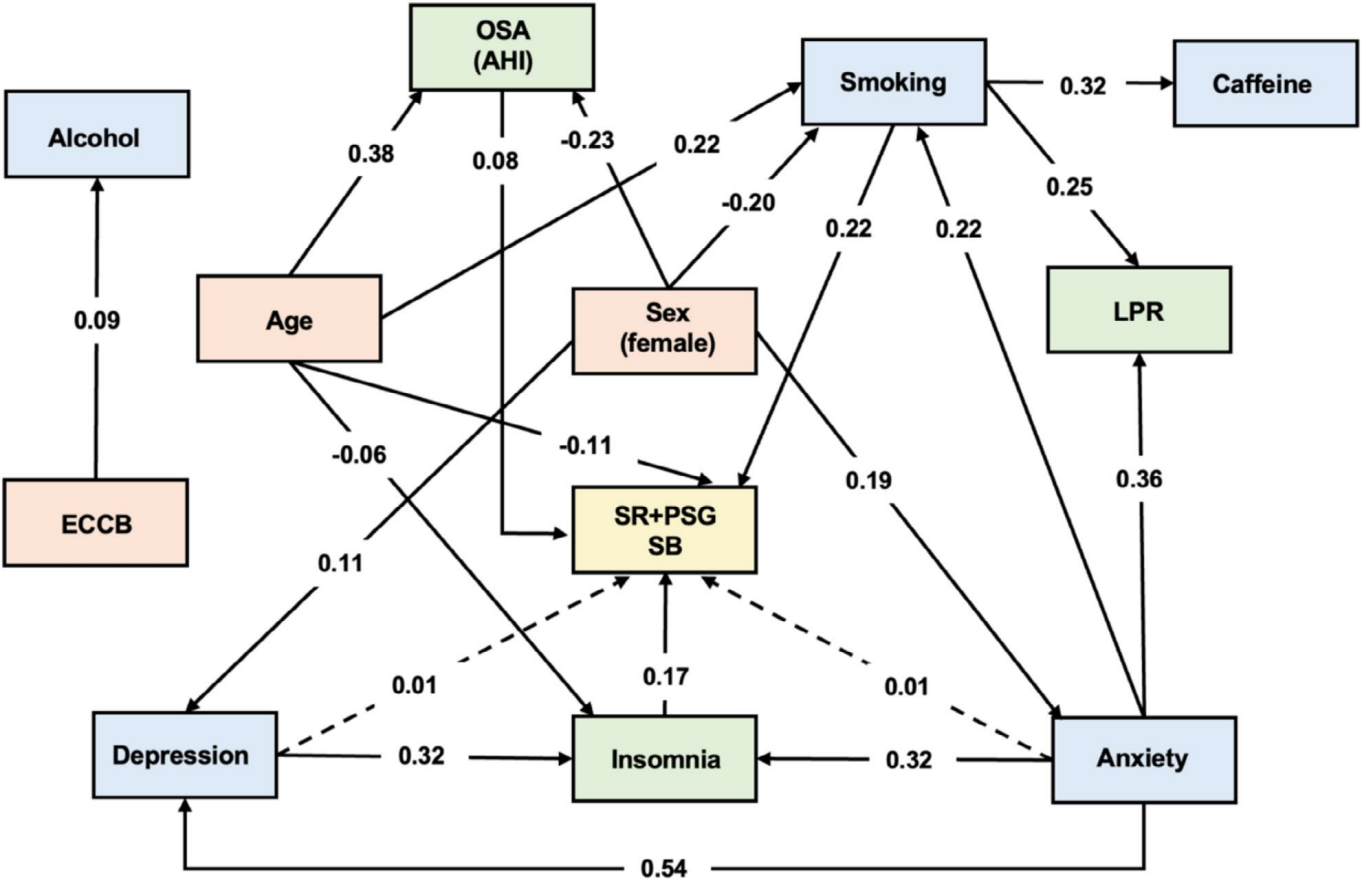
Significant direct and indirect pathways between predisposing factors and self‐report+PSG (SR + PSG) sleep bruxism (SB). Solid lines indicate direct pathways and dashed lines indicate indirect pathways (β values). AHI, apnea‐hypopnea index; ECCB, Economic Classification Criteria Brazil; LPR, laryngopharyngeal reflux; OSA, obstructive sleep apnea; SB, sleep bruxism.

Statistically significant direct and indirect pathways between predictor variables and sleep bruxism assessment by self‐report+PSG are shown in Figure 3. The occurrence of SB by self‐report+PSG was directly impacted by higher levels of OSA (β 0.08, SE 0.04, *p* < 0.01), insomnia (β 0.17, SE 0.04, *p* < 0.01) and smoking (β 0.22, SE 0.07, *p* < 0.01). Younger age (β −0.11, SE 0.04, *p* < 0.01) directly impacted SB by combined methods. Considering indirect effects, higher anxiety was associated with increased SB by self‐report+PSG via increased insomnia and LPR. Similarly, depressive symptoms (β 0.01, SE 0.01, *p* < 0.01) were indirectly impacted the outcome via increased insomnia. The model exhibited satisfactory fit indexes: RMSEA (0.01; 0.00–0.02), CFI (1.00), and TLI (1.00).

## 4 Discussion

This study aimed to investigate the pathways through which potential risk factors may influence sleep bruxism, as assessed by self‐report, PSG‐based, and a combination of these methods. Across both assessment methods, insomnia and younger age emerged as consistent direct predictors of sleep bruxism. Additionally, higher anxiety and depressive symptoms were indirectly associated with increased SB through elevated insomnia levels. Further associations were observed: self‐reported SB was directly influenced by higher socioeconomic levels, while PSG‐based and self‐report+PSG sleep bruxism assessment were directly impacted by increased obstructive sleep apnea and smoking. Furthermore, age was indirectly linked to PSG‐based SB through its effects on OSA and insomnia. These findings highlight both shared and specific pathways underlying SB depending on the assessment method, emphasising the complex interplay of sociodemographic, behavioural, psychological, and clinical factors involved in its aetiology.

Our results demonstrated that the main predictor associated directly with SB was high insomnia levels. Similarly, previous evidence suggested an association of SB with insomnia in different statistical approaches, such as logistic regression and correspondence analysis [14], as well as 2 studies with network analysis [15, 18]. The mechanisms that could be suggestive of an association between insomnia and SB could be the presence of arousals, which may be present in chronic insomnia, especially in maintenance insomnia [35], as well as in SB [36, 37]. Thus, individuals with insomnia often experience fragmented sleep and elevated physiological and cognitive hyperarousal, all of which can trigger or exacerbate RMMA during sleep, a characteristic feature of SB [35]. Insomnia has been linked to dysregulation of the hypothalamic–pituitary–adrenal (HPA) axis and increased sympathetic nervous system activity [38], which may contribute to the development or maintenance of SB episodes. Thus, although the available evidence on the association between insomnia and bruxism is limited and somewhat conflicting [39], our findings indicate that sleep fragmentation may play a key role in the manifestation of SB.

Younger age emerged as a consistent direct predictor of SB, a finding that aligns with previous epidemiological studies reporting higher prevalence rates of SB among younger individuals [7, 8]. This association may be explained by age‐related differences in sleep architecture, stress reactivity, and neurophysiological arousal. Adolescence and early adulthood are periods marked by heightened psychological stress and emotional regulation challenges [40], both of which have been implicated in the aetiology of SB. Over time, the prevalence of SB tends to decline, potentially due to neurophysiological adaptations or reduced responsiveness to environmental and internal stressors with age [7, 8, 41]. However, a recent study found that RMMA remains relatively stable over time, especially in the general population, suggesting that the association between RMMA index and age may not be absolute [21].

High levels of anxiety and depressive symptoms were indirectly associated with increased SB via elevated insomnia levels. This finding supports the biopsychosocial model of SB, which considers emotional and psychological factors as key contributors to their development and maintenance [15, 40, 42]. Anxiety and depression can negatively affect sleep quality and are associated with heightened cortical arousal, which may facilitate RMMA during sleep [43]. Insomnia may act as a mediating mechanism by increasing sleep fragmentation and nighttime awakenings, conditions that have been linked to greater SB activity [12, 35, 40], as previously discussed. Some studies suggest that anxiety may exert a more immediate influence on insomnia due to hyperarousal and worry‐related cognitive activity, whereas depression may contribute through disturbances in sleep architecture and circadian rhythms [43, 44]. Another factor to be considered is cortisol, the stress hormone, which is found in high levels in patients with insomnia [45] and is associated with anxiety and SB [42]. This distinction could imply slightly different underlying pathways for how each condition contributes to SB via insomnia. Our findings support the role of insomnia as a central pathway through which emotional distress influences SB.

Self‐reported SB was directly affected by higher ECCB scores, implying that a greater socioeconomic level impacts the higher occurrence of self‐reported SB. This finding may reflect increased health awareness and a greater probability of symptom recognition and reporting among individuals with higher socioeconomic levels. Previous studies have suggested that individuals from higher socioeconomic backgrounds may be more attentive to their health status and more likely to report sleep‐related symptoms, including bruxism [41, 46]. Moreover, a higher socioeconomic level is often associated with lifestyles involving greater psychological demands or stress, which may also contribute to increased SB [47], which may explain our findings.

Our findings showed that PSG‐based assessment of SB and the combination of methods (self‐report+PSG) appear to be directly influenced by high OSA levels. Previous studies have reported that 30%–50% of adults with OSA present SB, with more than 2 RMMA/h [17, 20]. However, the nature of the association between SB and OSA remains unclear. While it cannot be definitively established that OSA acts as a trigger for SB, both conditions may be regarded as overlapping comorbidities [48]. Although the causal direction between RMMA and apneic or hypopneic events has not been fully elucidated, some studies have demonstrated a temporal relationship that RMMA episodes are followed by respiratory events 3.7 to 10 times more frequently than the inverse [20, 49, 50, 51]. These findings cautiously reinforce the possible hypothesis that SB may be modulated by underlying sleep‐related breathing disorders such as OSA, highlighting the importance of a comprehensive sleep evaluation in patients presenting with suspected sleep bruxism.

The PSG‐based and the combination of methods (self‐report+PSG) were directly impacted by increased smoking. This association may be rationalised by the stimulating effects of nicotine on the CNS and its role in increasing arousal frequency during sleep, which can trigger SB episodes [12]. Still, smoking has been linked to poorer sleep quality and higher prevalence of sleep disturbances, both of which are associated with SB [41, 52]. Age was indirectly associated with PSG‐based SB through its effects on OSA and insomnia. Previous studies have depicted that advancing age increases the risk of sleep apnea due to upper airway changes and that insomnia symptoms are also more common in older adults [53, 54]. These sleep disturbances can lead to increased arousals during sleep, which are known triggers for SB episodes [37], which justify current findings.

This study has some limitations that should be acknowledged. Its cross‐sectional design limits causal inferences, especially considering pathway analysis. In recent years, we have seen a growing interest in Sleep Medicine and greater access to information. The use of self‐reported measures may be subject to recall or reporting bias. The fact that this sample is a follow‐up from the initial sample may have led to an increase in SB prevalence, since from the initial sample, 66% who returned for the follow‐up were possible individuals with sleep complaints. Moreover, individuals who are aware of their SB may also be more attuned to its potential negative effects on their sleep quality, daily performance, and overall well‐being. It should be acknowledged that certain medical conditions may progressively worsen with age, potentially influencing the manifestation of SB. Furthermore, while PSG provides an objective and standardised assessment, bruxism events exhibit considerable night‐to‐night variability—even among individuals with frequent complaints—indicating that a single‐night recording may not fully represent the individual's habitual SB pattern. Although a range of relevant variables was included, other unmeasured variables may also influence SB. Thus, factors such as medication use, history of trauma, chronic diseases, and awake bruxism are important factors to be used in future studies.

Despite these limitations, the study has numerous strengths, including the use of both subjective and objective SB measures, a population‐based sample, and the application of SEM to explore complex pathways. The inclusion of a broad range of potential risk factors also enhances the comprehensiveness and relevance of the findings. Future longitudinal studies are encouraged to confirm these pathways, explore other causal mechanisms and consider the circadian manifestations of bruxism.

## 5 Conclusions

Our results indicated potential risk factors for SB assessed by self‐report, PSG‐based and combined methods. Insomnia and younger age consistently emerged as direct predictors of SB in all outcomes evaluated. Higher socioeconomic status was associated with self‐reported SB, while PSG‐based SB and combined methods were influenced by obstructive sleep apnea and smoking. Considering indirect pathways, anxiety and depressive symptoms influenced SB via elevated insomnia levels. These findings reinforce the multifactorial nature of SB and the importance of considering different strategies for its management.

## Funding

This work was supported by Associação Fundo de Incentivo à Pesquisa, Coordenação de Aperfeiçoamento de Pessoal de Nível Superior, Conselho Nacional de Desenvolvimento Científico e Tecnológico, Fundação de Amparo à Pesquisa do Estado de São Paulo.

## Conflicts of Interest

The authors declare no conflicts of interest.

## Data Availability

The data that support the findings of this study are available on request from the corresponding author. The data are not publicly available due to privacy or ethical restrictions.
